# Foxp3^+^ Regulatory T Cells among Tuberculosis Patients: Impact on Prognosis and Restoration of Antigen Specific IFN-γ Producing T Cells

**DOI:** 10.1371/journal.pone.0044728

**Published:** 2012-09-19

**Authors:** Amar Singh, Aparajita Ballave Dey, Anant Mohan, Prabhat Kumar Sharma, Dipendra Kumar Mitra

**Affiliations:** 1 Cellular Immunology Division, Department of T.I.I., All India Institute of Medical Sciences, Ansari Nagar, New Delhi, India; 2 Department of Medicine, All India Institute of Medical Sciences, Ansari Nagar, New Delhi, India; New York University, United States of America

## Abstract

CD4^+^CD25^+^Foxp3^+^ Regulatory T cells (Treg) and programmed death-1 (PD-1) molecules have emerged as pivotal players in immune suppression of chronic diseases. However, their impact on the disease severity, therapeutic response and restoration of immune response in human tuberculosis remains unclear. Here, we describe the possible role of Treg cells, their *M. tuberculosis* driven expansion and contribution of PD-1 pathway to the suppressive function of Treg cells among pulmonary tuberculosis (PTB) patients. Multicolor flow cytometry, cell culture, cells sorting and ELISA were employed to execute the study. Our results showed significant increase in frequency of antigen-reactive Treg cells, which gradually declined during successful therapy and paralleled with decline of *M. tuberculosis*–specific IL-10 along with elevation of IFN-γ production, and raising the IFN-γ/IL-4 ratio. Interestingly, persistence of Treg cells tightly correlated with MDR tuberculosis. Also, we show that blocking PD-1/PD-L1 pathway abrogates Treg-mediated suppression, suggesting that the PD-1/PD-L1 pathway is required for Treg-mediated suppression of the antigen-specific T cells. Treg cells possibly play a role in dampening the effector immune response and abrogating PD-1 pathway on Treg cells significantly rescued protective T cell response, suggesting its importance in immune restoration among tuberculosis patients.

## Introduction

A strong IFN-γ biased Th1 effector response is critical for immune containment of tuberculosis. Several recent reports including ours demonstrate the presence of Th-1 like response, in localized form of disease while its deficit tightly correlates with disseminated disease [1], [2]. Proportional presence of various T cell subsets is believed to dictate the bulk T cell response determining the host immune status [3], [4]. Regulatory T cells (Treg) have been implicated in suppressing the effector T cell response among tuberculosis patients [5], [6], [7]. Treg cells suppress the immune response both via contact dependent and independent mechanisms [8], [9], [10]. Contact dependent suppression by the Treg cells is mediated by generation of immunosuppressive molecule, adenosine by ectopeptidases, membrane bound TGF-β and expression of inhibitory molecules such as PD-1. Programmed Death-1 (PD-1) has been recently identified as an immune exhaustion marker inhibiting effector T cell response [11], [12], [13]. Role of interaction between PD-1 with its ligands in dampening of T cell response has recently been observed in infectious diseases like HIV [14], [15], HCV [16], [17], [18] and tuberculosis [19], [20]. Recently PD-1 mediated suppression of allospecific T cells by Treg cells has been identified [21], [22]. However, studies on the importance of PD-1 molecules in Treg mediated immunosuppression in infectious diseases are scanty, even though higher levels of PD-1 expression on these cells are reported [23], [24]. Previously we observed enrichment of Treg cells among tuberculosis patients and their role in suppression of effector T cell response via Interluekin-10 (IL-10) [5]. However, the precise role of PD-1 in causing suppression of effector T cell response is yet to be deciphered. Here, we report the critical role of Treg cells in the suppression of effector response in human tuberculosis and rescuing the as antigen specific IFN-γ producing T cells. We investigated frequency dynamics, functionalities of Treg cells among pulmonary tuberculosis (PTB) patients and its relationship with *M. tuberculosis* (*Mtb.*) specific effector cytokine production. To reveal the impact of PD-1 expressed by Treg cell on the protective T cell response, blocking studies were performed on the PBMCs obtained from PTB patients. Our data reveals that peripheral representation of Treg cells tightly correlates with the disease severity and decline after successful therapy, which parallels with rise in *Mtb.* specific protective cytokine response. However, failure in Treg cells decline during therapy was tightly associated with multi-drug resistances (MDR) tuberculosis. Abrogating Treg cells could rescue significantly the *Mtb.* specific Th-1 response. Our findings strongly indicate possible role of PD-1 expressed on Treg cells in the pathogenesis of human tuberculosis and provide molecular insights in to their suppressive effect. This pathway may prove an important target for restoring host protective immunity.

## Results

### Enrichment of CD4^+^CD25^+^Foxp3^+^ Treg cells in the peripheral blood of PTB patients

**Figure 1:**
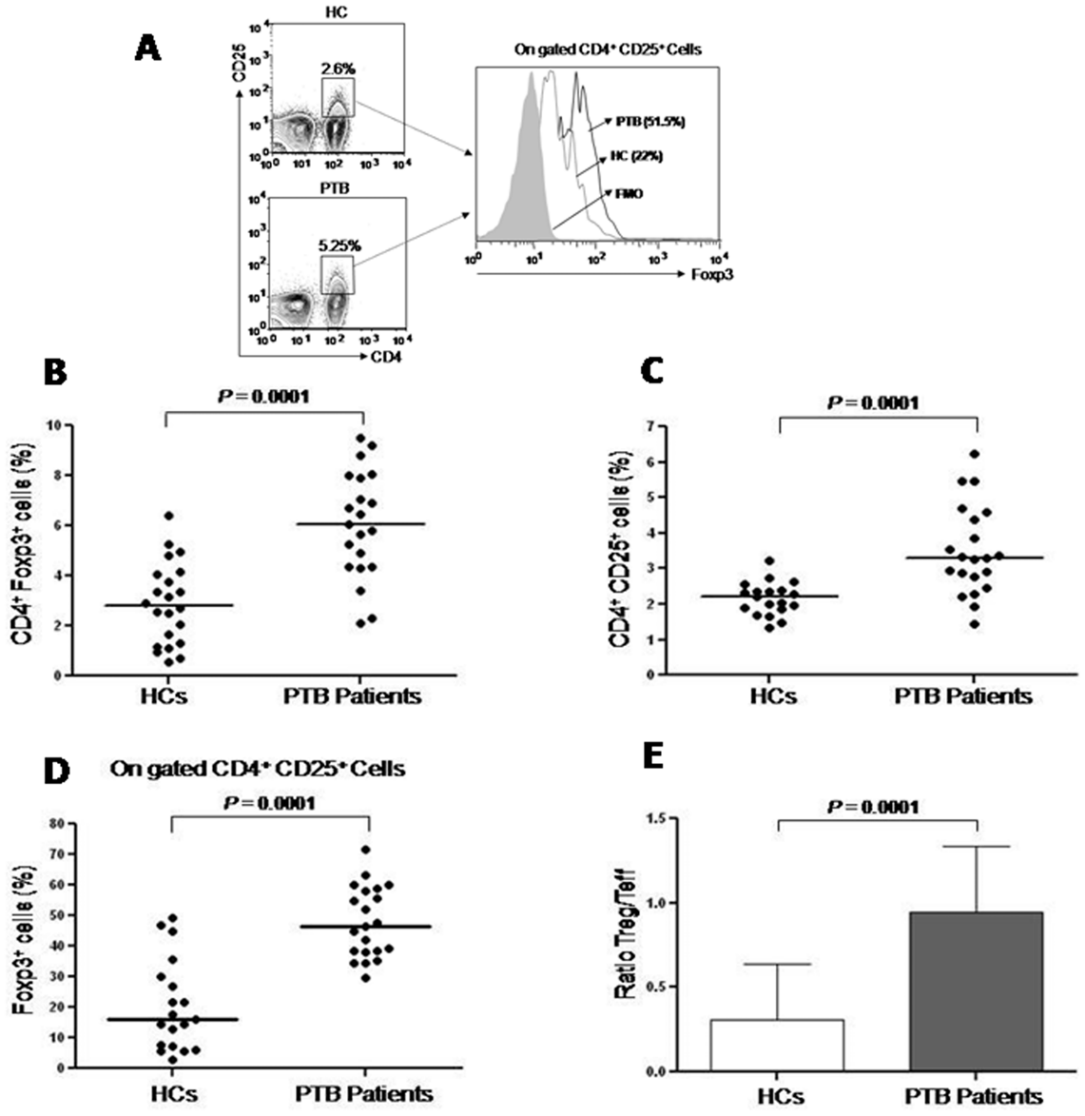
CD4^+^CD25^+^Foxp3^+^ Treg cells frequencies are increased in the peripheral blood of PTB patients. PBMCs isolated from the peripheral blood of Pulmonary Tuberculosis Patients (PTB, n = 21) and Healthy Controls (HCs, n = 19) were analyzed for surface expression of CD4 and CD25 and intracellular expression of Foxp3. CD4^+^CD25^+^ T cells were gated for analysis of Foxp3 expression. (**A**) Histograms (shaded grey-Fluorescence Minus One (FMO); dotted line-HCs and smooth line-PTB patients) showing Foxp3 expression. (**B**) Percentage of Foxp3 cells gated on CD4^+^ T cells. (**C**) Percentage of CD4^+^CD25^+^ cells on gated lymphocytes. (**D**) Percentage of Foxp3^+^ cells within gated CD4^+^CD25^+^ cell population (**E**) Ratio of Treg and Teffector cells in peripheral blood of PTB patients and HCs. Statistical analyses of values between cell population from PTB patients and Healthy Controls were performed with the nonparametric Mann Whitney U test, two-tailed for unpaired data. Each symbol represents a single individual.

We enumerated Treg on the basis of single cell level expression of CD4^+^CD25^+^Foxp3^+^ in PBMCs isolated from the peripheral blood of PTB patients (n = 21) and Healthy Controls (HCs, n = 19) subjects. Peripheral frequencies of Treg cells and the ratio of Treg cells vs. non Treg (mixture of Teff and resting T cells in various proportions) were significantly higher among PTB patients than that of HCs. (**Fig. 1A, B, C, D and E**). Enrichment of Treg cells among PTB patients may be constitutive or due to their *Mtb.* induced expansion.

### Correlation between Foxp3^+^ Treg cells with bacillary load and Mtb. specific immune response among PTB patients

**Figure 2:**
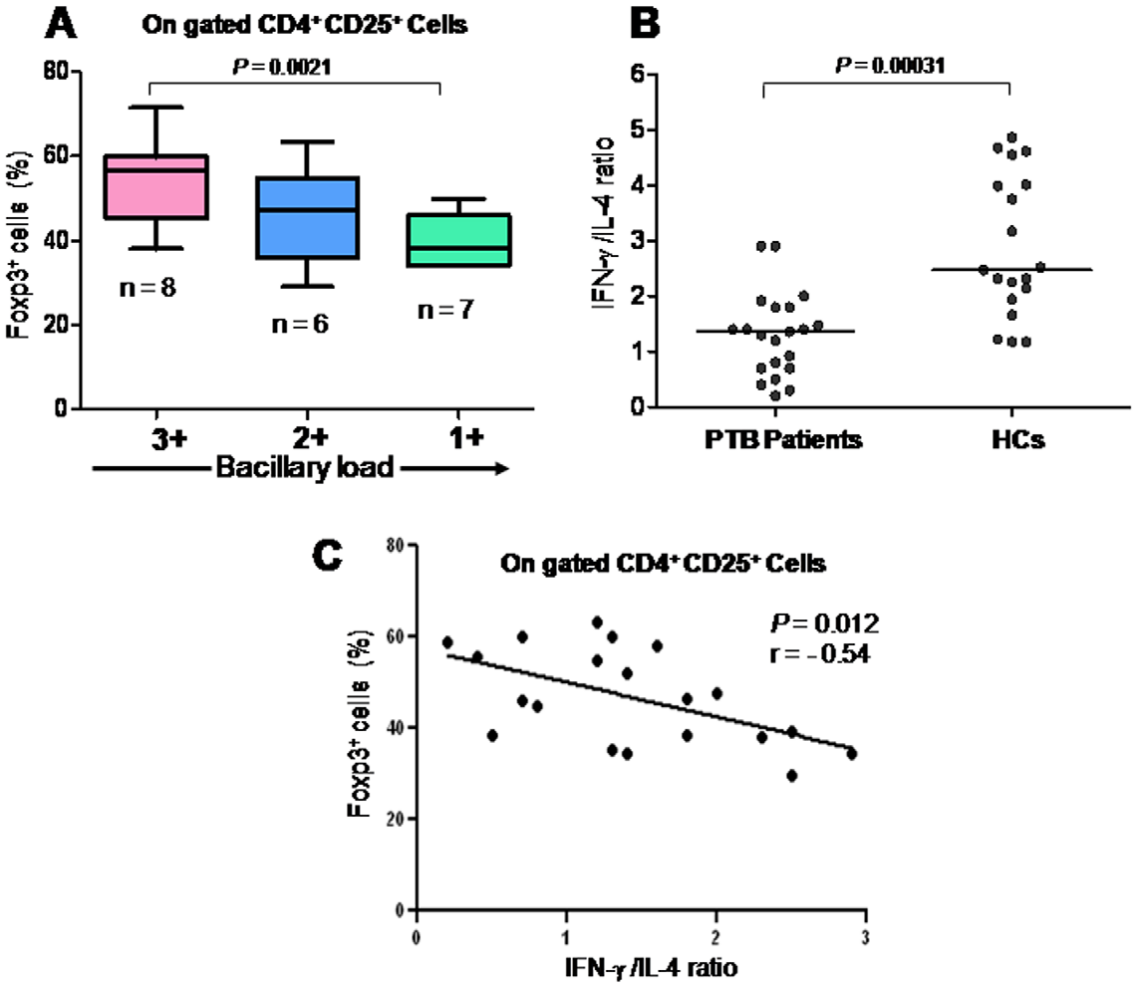
Increased frequency of CD4^+^CD25^+^Foxp3^+^Treg cells positively correlates with bacillary load in the sputum of PTB patients and inversely with *Mtb.* antigen specific cytokines response in PTB patients. Percentage of Treg cells from freshly isolated PBMCs of PTB patients correlated with the bacillary load among the patients. (**A**) CD4^+^CD25^+^Foxp3^+^ Treg cells frequencies were significantly higher among patients with high bacillary load (3+) than those with lower bacillary load (1+). Statistical analyses of values between cell population from 3^+^ (n = 8), 2^+^ (n = 6) and 1^+^ (n = 7) bacillary load were performed with the nonparametric Mann Whitney U test for unpaired data. (**B**). PBMCs isolated from PTB patients were cultured for 24 hrs with *Mtb.* antigen (WCL). Cultured cells were washed and surface stained with anti-CD4 followed by IFN-γ and IL-4 intracellular staining. Statistical analyses of ratio of IFN-γ and IL-4 producing CD4 T cells from PTB patients and HCs were performed with the nonparametric Mann Whitney U test, two-tailed for unpaired data. Each symbol represents a single individual. (**C**) Inverse relationship between CD4^+^CD25^+^Foxp3^+^ Treg cells and *Mtb.* antigen specific IFN-γ and IL-4 ratio from PTB patients (n = 21; correlation statistics were analyzed using the Spearman correlation).

We observed a tight correlation between the bacillary load and Treg cells frequency in the peripheral blood of treatment naïve PTB patients (**Fig. 2A**). This indicates antigen induced Treg cell generation and the role of *in vivo* pathogen exposure (load) in this process. It may also be possible that increased bacillary load leads to increased inflammation and a subsequent expansion of Treg cells during active tuberculosis as shown by Green et al [25]. We also evaluated effector T cell function in terms of ratio of antigen specific IFN-γ and IL-4 producing CD4 T cells following *in vitro* stimulation (**Fig. S1**). Significantly, lower ratio among PTB patients compared to that of HCs indicates a suppressed state of effector T cell response among PTB patients (**Fig. 2B**). Moreover, an inverse correlation was present between the Treg cell frequency and IFN-γ/IL-4 ratio (effector T cell function) among PTB patients (**Fig. 2C**). All together, our data suggests that enriched Treg cells plausibly suppress the antigen specific protective cytokine (IFN-γ) response.

### *Mtb.* driven expansion of Treg cells and diseases severity

**Figure 3:**
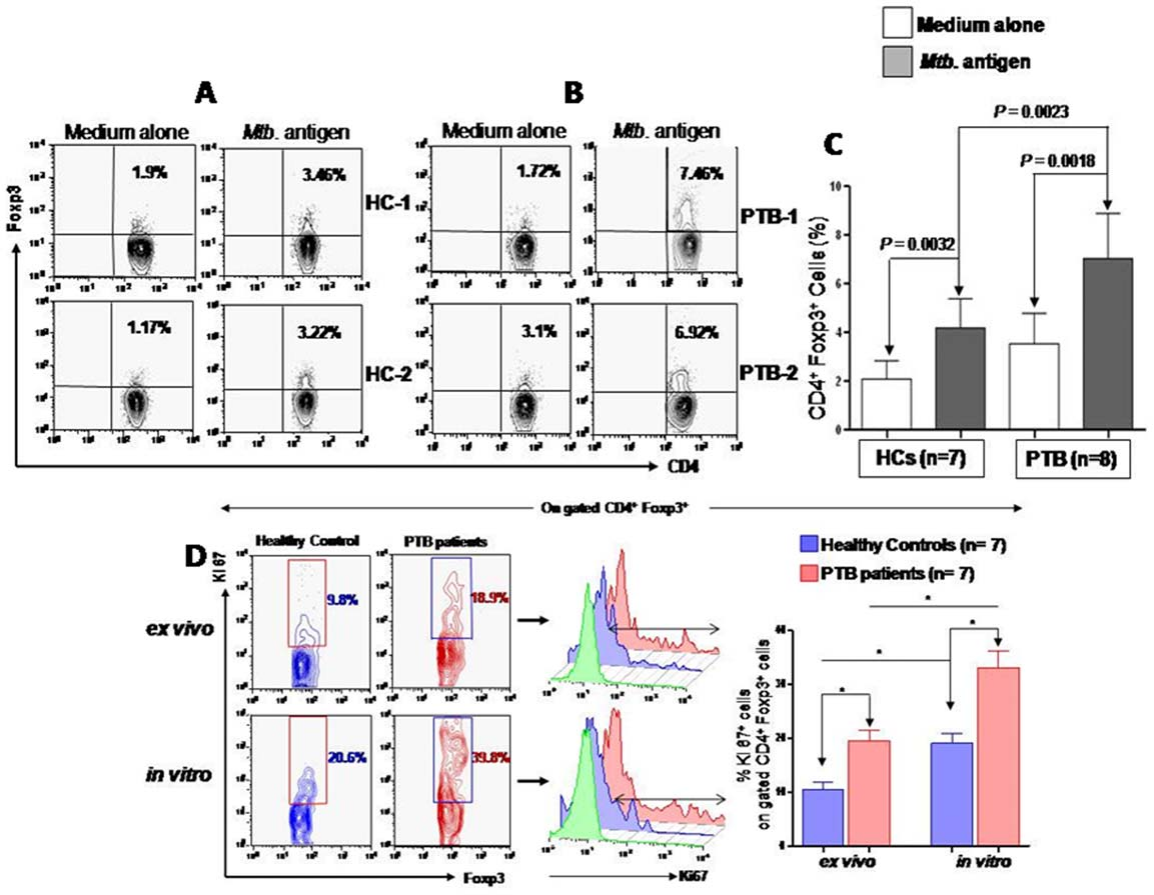
*Mtb.* antigen specific expansion of CD4^+^CD25^+^Foxp3^+^Treg cells. PBMCs isolated from PTB patients and HCs were cultured for 48 hours with or without *Mtb.* antigen (WCL). Cultured cells were washed and surface stained with anti-CD4 followed by intracellular Foxp3 staining. (**A–B**) Representative FACS plot showing frequencies of Treg cells with or without antigenic stimulation in PBMCs of both HCs and PTB patients (**C**) Statistical analyses of values between the percentages of CD4^+^Foxp3^+^ cells between unstimulated and *Mtb.* antigen (WCL) stimulated, were performed with Wilcoxon rank sum test for paired samples. Values were shown in mean ± S.D. for HCs (n = 7) and PTB patients (n = 8). (**D**) Percentage of Ki67^+^ cells analyzed by flow cytometry both *ex vivo*: freshly isolated PBMCs and *in vitro*: PBMCs pulsed with *Mtb.* antigen (WCL) for 48 hrs in peripheral CD4^+^Foxp3^+^ cells population both in HCs and PTB patients. Proliferating cells (Ki67^+^) were significantly higher in freshly and *Mtb.* stimulated CD4^+^Foxp3^+^ cells population of PTB patients than HCs. Statistical analysis were performed with Wilcoxon rank sum test for paired samples.

To investigate if the enrichment of Treg cells results from *Mtb.* induced expansion, we evaluated both the *ex vivo* as well as in vitro proliferation of Treg cells on the freshly purified PBMCs (a measure of Treg cell expansion in response to *in vivo* pathogen/antigen exposure) and after antigenic stimulation. Following *in vitro* antigen stimulation the frequency of Foxp3^+^ Treg cells increased significantly and markedly among PTB patients compared to that of HCs (**Fig. 3 A, B and C**). To further investigate the possibility of antigen induced expansion of Treg cells among PTB patients, we evaluated their fraction in the proliferative cycle in response to mycobacterial antigens by enumerating Ki67^+^ cells within gated Foxp3^+^ population. We observed significantly higher fraction of proliferating Treg cells *ex vivo*, suggesting their expansion by the *Mtb.* infection during the disease pathogenesis. This is further supported by markedly higher antigen induced *in vitro* proliferation of Treg cells (**Fig. 3D**).

### Treg cells and antigen specific effector T cell response among PTB patients

**Figure 4:**
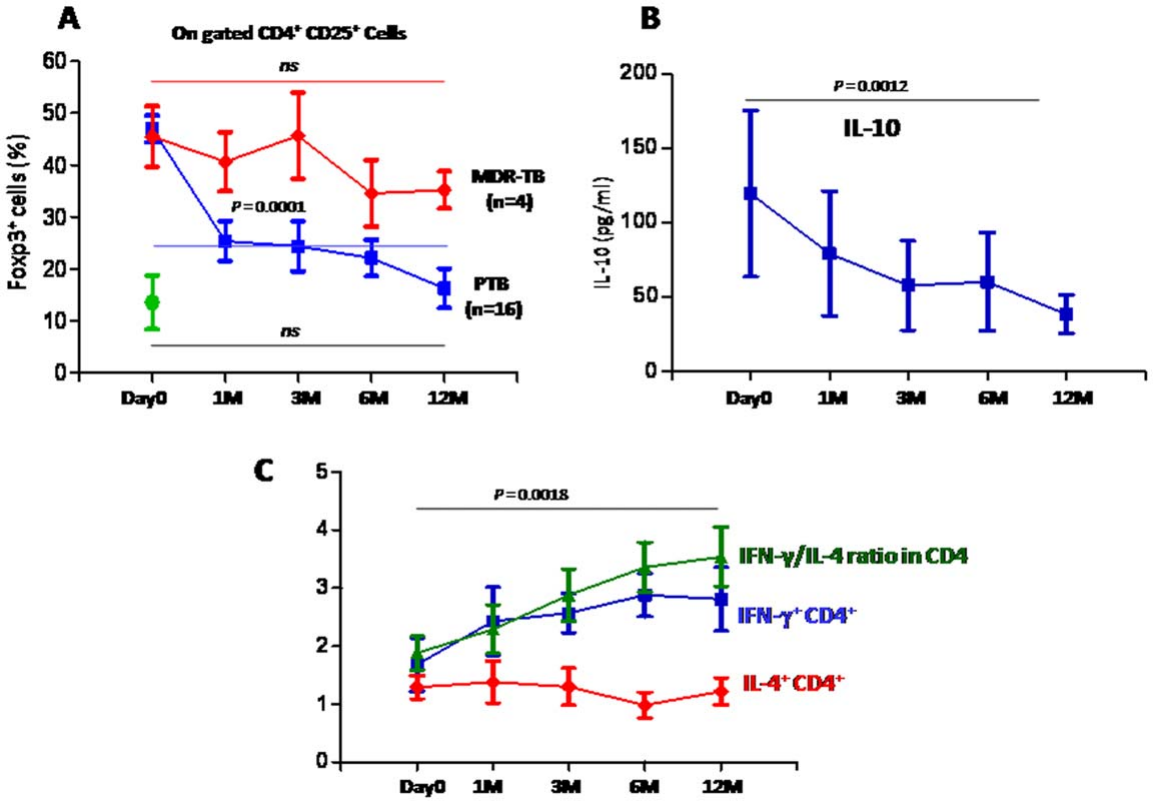
The frequency of CD4^+^CD25^+^Foxp3^+^ Treg cells decline after successful anti-tuberculosis therapy. At each time point freshly isolated PBMCs from PTB patients were analyzed for CD4^+^CD25^+^Foxp3^+^ Treg cells frequency. (**A**) The percentage of CD4^+^CD25^+^Foxp3^+^ Treg cells was determined in 16 PTB patients (▪ line) 4 MDR TB (♦ line) at various time point (Day0-before commencement of therapy and at 1 month, 3 month, 6 month and 12 month) and in 19 HCs. (•) at day 0. (**B**) Decline in Treg cells frequency correlated with soluble IL-10 levels secreted in culture supernatants of *Mtb.* antigen (WCL) stimulated PBMCs. At each time point freshly isolated PBMCs from PTB patients were cultured with *Mtb.* antigen (WCL) for 24 hrs and culture supernatants were utilized for IL-10 ELISA. IL-10 levels significantly declined with time and correlates with decline in Treg cells frequency. (**C**) PBMCs from PTB patients cultured with *Mtb.* antigen (WCL) for 24 hrs and intracellular cytokines (IL-4 and IFN-γ) and their ratio determined for CD4^+^T cells by flow cytometry. Along the course of treatment, we observed significant decline in Treg cells frequency with significant increased in IFN-γ/IL-4 ratio among these patient. Increased in IFN-γ/IL-4 ratio is primarily due to increased in the frequency of *Mtb.* antigen specific IFN-γ producing CD4 T cells.

We analyzed the relationships between Treg cell enrichment and *Mtb.* specific cytokine (IFN-γ and IL-4) producing helper T cells among PTB patients (n = 21) who underwent anti-tuberculosis therapy and followed longitudinally. (**Fig. 2B** **and** **Fig. 4A**). Previously, we have demonstrated the key role of IL-10 in Treg mediated suppression of Teff cells [5]. Thus, we also estimated the IL-10 levels in culture supernatant harvested at 24 hours of *Mtb.* specific culture of PBMCs obtained from PTB patients. Interestingly, we found gradual and significant decline of CD4^+^CD25^+^Foxp3^+^Treg cells during anti-TB therapy and at 12 month their percentages came down to the levels comparable with HCs (**Fig. 4A** **,** **Fig. S2**). We also observed tight correlation between the decline in the frequency of Treg cells and reduction in the levels of soluble IL-10 produced by the bulk T cells *in vitro* (**Fig. 4A, B** **, Fig. S3**). We envisaged that the dynamics of Treg cells and IL-10 produced by them play important role in suppressing the host protective T cell response. This is further substantiated by significant increase of *Mtb.* specific IFN-γ producing T cells during successful therapy (**Fig. 4C**) increasing IFN-γ/IL-4 ratio, indicating a Th-1 shift of host immunity. This shift of effector T cell response towards protective response during therapy was primarily due to restoration of IFN-γ production, as no significant changes was noted for IL-4 production in response to *Mtb.* antigen during the follow up (**Fig. 4C**). This correlated well with the improvement in the clinical parameters (Chest X-ray and bacillary index). We were intrigued to note that patients who failed to show significant decline in the Treg cells frequency during therapy were unresponsive to anti-tubercular therapy and subsequently diagnosed as multi-drugs resistance (MDR) cases (**Fig. 4A**). Failure of decline in Treg cell frequency among MDR-TB cases is indicative of Treg cell expansion by persistent pathogen load. This expansion may be driven by the chronic inflammatory state of tuberculosis and pathogen itself or both. On the other hand decline in Treg cell frequency may arise due to clearance of bacilli resulting in waning of antigens and thus subsequent reduction in their frequencies. Another possibility is that during active tuberculosis high bacterial burden may be a causal factor for Treg expansion leading to the continues weakening of host immune response thus providing prolong favorable environment for bugs and the same time drug induce stress causing development of resistance against anti-tubercular drugs.

### Reversal of Treg mediated suppression by blocking PD-1-PD-L1 pathway

**Figure 5:**
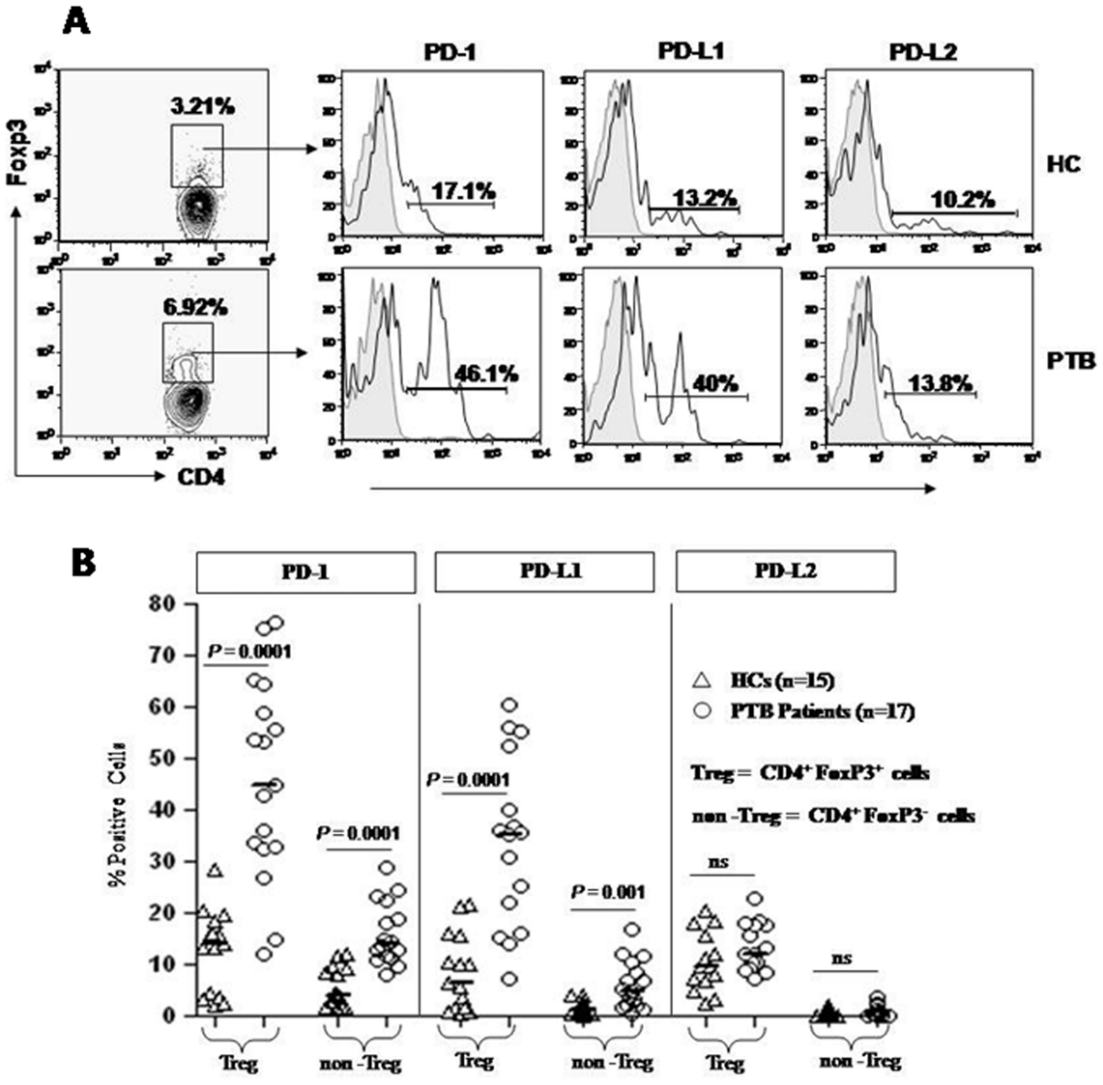
Up regulation of PD-1 on CD4^+^Foxp3^+^ Treg cells from PBLs of PTB patients. PBMCs isolated from PTB patients and HCs were surface stained with anti-CD4 and anti-PD-1, anti-PD-L1 and anti-PD-L2 followed by Foxp3 intracellular staining. (**A**) Representative histogram shows analysis of PD-1 expression on CD4^+^Foxp3^+^ cells populations among HCs and PTB patients. (**B**) Percentages of PD-1, PD-L1 and PD-L2 on CD4^+^Foxp3^+^ Treg cells and CD4^+^Foxp3^−^ non Treg cells populations from PBLs of PTB patients and HCs. Statistical analyses of values between PTB and HCs were performed with the nonparametric Mann-Whitney U test, two-tailed for unpaired data. Each symbol represents a single individual.

Recent demonstration of preferential expression of PD-1 and its ligands on T cell subsets in various chronic pathologic conditions [11], [12], [13], [14], [15], [16], [17], [18], [19], [20] and important role of up-regulated PD-1 on Treg in immunosuppression [22], [24] encouraged us to evaluate it's expression on Treg cells and possible impact on *Mtb.* specific host immune response. We examined the expression of PD-1 and its ligands on regulatory (CD4^+^ Foxp3^+^) and effector T cells (CD4^+^Foxp3^−^) in PBMCs of PTB patients. We observed significantly higher frequencies of PD-1^+^ and PD-L1^+^ Treg cells among PTB patients (**Fig. 5A, B**). Similar trend of PD-1 and PD-L1 expression, though of much lower magnitude was also observed on the non-Treg cells of PTB patients. PD-L-2 expression was similar both in patients as well as HCs (**Fig. 5A, B**). Perhaps this is the first report on the expression of PD-1 ligands on Treg cells among PTB patients. These results inspired us to investigate the possible suppressive role of PD-1-PD-L1 pathway in dampening *Mtb.* specific effector T cell response.

**Figure 6:**
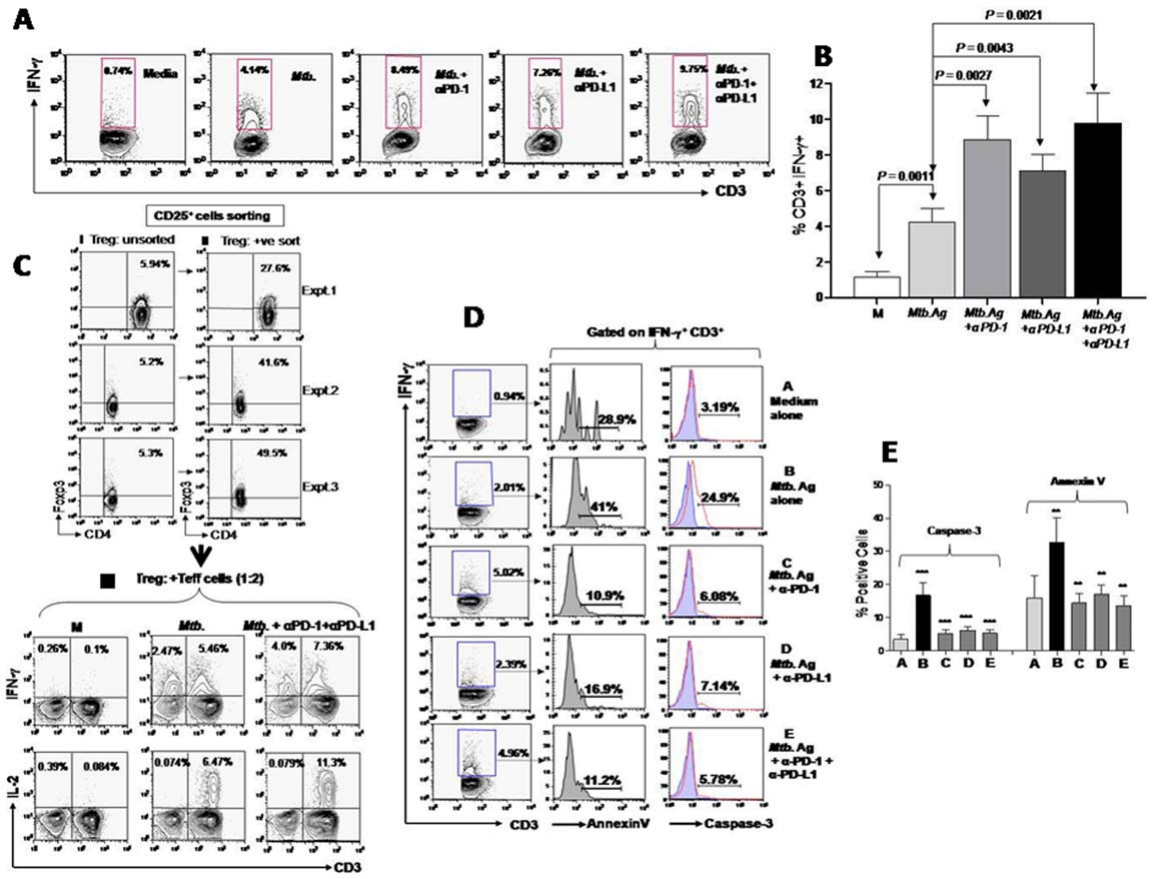
*In vitro* blocking effect of PD-1-PD-L1 interactions on the effector T cell functions and survival among PTB patients. PBMCs from tuberculosis patients were stimulated with *Mtb.* antigen (WCL) in the presence or absence of anti-PD-1 mAb, anti-PD-L1 and anti-PD-1+PD-L1 mAb for 72 hrs followed by intracellular detection of IFN-γ by flow cytometry. Lymphocytes were gated based on their scatter (FSC/SSC) profile and further gated on CD3^+^ T cells. (**A**) Representative FACS plots shows production of IFN-γ by CD3^+^ T cells which markedly increases upon blocking PD-1-PD-L1 interaction (**B**) Each histogram depicts the effect of PD-1, PD-L1 blocking on the percentage of IFN-γ^+^ CD3^+^ T cells (mean ± S.E.M. for n = 8). (**C**) Enriched Treg cells (Foxp3^+^) were obtained by magnetic sorting of CD25^+^ cells. These cells were treated with anti-PD-1+PD-L1 blocking antibody and subsequently added to T cells enriched population in ratio of 1∶2 (Treg Vs Teff) with or without *Mtb.* antigen (WCL). Blocking PD-1 and PD-L1 markedly increases the frequency of IFN-γ and IL-2 producing T cells (I, II and III). Data is representative of three individual experiments. (**D**) *In vitro* blocking effect of PD-1-PD-L1 interactions on survival of effector T cells. PBMCs from PTB patients were stimulated with *Mtb.* antigen (WCL) for 72 hrs in the presence or absence of anti-PD-1 mAb, anti-PD-L1 and anti-PD-1+PD-L1 mAb and intracellular IFN-γ expression was determined by flow cytometry. Further annexin V and caspase-3 positivity was determined on effector T cells (IFN-γ^+^ CD3^+^). Blocking PD-1-PD-L1 interactions significantly decreases the apoptosis of *Mtb.* specific IFN-γ producing T cells as determined by expression of annexinV and caspase-3. (**E**) The pooled data represents the mean ± S.D. performed on 3 PTB patients in duplicates.** P≥0.001 and *** P≥0.0001.

Given that the production of IFN-γ by T cells is critical for immunity against *Mtb.* infection, we evaluated the effect of PD-1-PD-L1 blockade on the IFN-γ production by T cells in response to *Mtb.* antigen *in vitro*. Interestingly, blocking PD-1 and PD-L1, either alone (8.49% and 7.26% respectively) or together (9.75%) markedly increased the percentage of antigen specific IFN-γ producers (**Fig. 6A, B**), suggesting the suppressive role of this pathway in dampening the specific T cell response among PTB patients. Interestingly, blocking of PD-1 expressed on Treg cells abrogated their antigen specific suppressive activity and significantly increased the frequency of effector IFN-γ and IL-2 producing T cells (**Fig. 6C** **, III upper and lower panel**). These findings strongly indicate that PD-1 and PD-L1 expressed on Treg cells, at least in part are involved in dampening the effector function of antigen reactive T cells among PTB patients. Moreover, IFN-γ production by non-T cells could also be restored, suggesting that Treg cells possibly suppress the NK cells (CD3^−^), which are known to be the early innate producers of IFN-γ. However, our result doesn't exclude the role of PD-1, PD-L1 expressing non-Treg cells (CD4^+^Foxp3^−^) in suppression of host effector T cell response. However, marked and preferential expression of these molecules on Treg cells provides definitive evidence for the role of PD-1-PD-L1 pathway in the Treg cell mediated suppression of specific Teff cell function among PTB patients.

To investigate if the suppressive influence of PD-1-PD-L1 pathway on the antigen specific IFN-γ producing effector T cells is due to induction of apoptosis, we evaluated the expression of annexinV, an early pre-apoptotic marker and caspase-3 expression, on the antigen reactive IFN-γ producing T cells of PTB patients. *Mtb*. specific IFN-γ producing T cells showed that almost half of them underwent apoptosis (41% annexinV^+^, and 24.9% caspase-3^+^ (**Fig. 6D** **, middle and right panel**). Intriguingly, blocking PD-1 and PD-L1, alone or together, markedly reduced the proportion of apoptotic IFN-γ producing T cells (10.9%, 16.9% and 11.2% annexinV^+^ respectively and 6.08%, 7.14% and 5.78% caspase-3^+^ respectively) from PTB patients (**Fig. 6E**). This clearly indicates that PD-1-PD-L1 pathway induces apoptosis of *Mtb.* specific effector T cells. Importantly, this suppressive influence of Treg and PD-1, PD-L1 expressed on them could be abrogated and may result in restoration of protective effector T cell function among PTB patients. Taken together, present data suggest that PD-1 and PD-L1 expressed on Treg cells also participate in dampening of type 1 immune response through apoptosis of IFN-γ producing T cells.

## Discussion

Protective immunity against *Mtb.* critically involves IFN-γ biased Th-1 effector immune response [26], [27], that influences the varied clinical manifestations of human tuberculosis [1]. During active tuberculosis, suppression of *Mtb.* specific T cell response is evidenced by decreased production of the cytokines such as IL-2 [28] and interferon IFN-γ [29]–[31] with simultaneous over production of suppressive cytokines such as IL-10 [32] and TGF-β [33]. Recently we and others have demonstrated that Foxp3^+^ regulatory T cells are over represented in tuberculosis and suppress the host effector T cell response against *Mtb.* particularly at the pathologic site of disseminated tuberculosis [5], [32]–[33]. Kursar et al demonstrated the suppression of *Mtb.* specific effector T cell function by Treg cells in Rag1^−/−^ mice. [34]. Recent studies indicate IL-10 dependent Treg cell mediated suppression of host immunity [5], [9] and higher expression of immune exhaustion marker PD-1 in tuberculosis [19], [20]. PD-1 is also known to be preferentially expressed on Treg cells [35]. However, precise role of Treg cells and PD-1 expressed on them in suppression of host T cells response against *Mtb.* among patients is yet to be unraveled.

Here, we demonstrate marked enrichment of Foxp3^+^ Treg cells in the peripheral blood of PTB patients. Ratio of Treg/Teff cells tilted towards the former suggesting the relative dominance of Treg cells. We revealed higher level of such enrichment among patients with high bacillary load and that inversely correlated with the pathogen specific IFN-γ skewed effector T cell response as measured by ratio of IFN-γ/IL-4 (**Fig. 2B**). On follow up after chemotherapy, decline of Treg cells and IL-10 along with restoration of *Mtb.* specific IFN-γ production, suggest Treg cell mediated active suppression of host immunity. Several recent studies on mice and human indicate Treg cell expansion during tuberculosis [5], [7], [36]–[39] and viral infection [35], [40]. Higher frequency of Treg cells among patients with higher bacillary load strongly hints towards the antigen induced Treg cells generation among patients. Similar observation has recently been reported among patients with visceral leishmaniasis [41]. Although several literatures [5], [7], [36]–[39] support an association between active disease and increased frequencies of Treg cells in the blood of humans, in contrary to this, recent work on non human primate showing that increased frequencies of Treg cells in active disease occur in response to increased inflammation [25]. Data obtained from this study suggest that the presence of more Treg cells before infection correlates with a better infection outcome. Burl et al observed lower number of Treg cells among latent tuberculosis patients (possibly due to their sequestration in the disease site) which reappeared during active tuberculosis, suggesting important role of Treg cells in human tuberculosis. [39]. According to these authors the increased Treg cell frequency observed in the blood of people with active tuberculosis is likely a response to inflammation and bacterial burden. Previously, we and others have demonstrated the suppression of *Mtb.* specific Th cell response by Treg cells in human [5], [7], [36]–[39] as well as in mice [6], [34]. Here, we show that a sizable fraction of the Treg cells in patients are reactive to *Mtb.* Therefore, we believe that in addition to inflammation, *Mtb.* may also induce expansion of antigen specific Treg cells, thus contributing to the observed Treg cell enrichment among PTB patients (**Fig. 3D**).

Our data of tight correlation between the MDR status and persistence of Treg cells suggest the important role of *Mtb.* driven expansion and maintenance of Treg cells in human tuberculosis. This is further supported by the antigen reactivity of the enriched Treg cells (**Fig. 3D**). It is plausible that during active tuberculosis high bacterial burden may be a causal factor for Treg expansions leading to the continued weakening of host immune response thus providing prolong and favorable milieu for emergence of MDR strains of bacilli. In any case, our result indicates the importance of Treg cell as suggestive biomarker for MDR form of tuberculosis.

All together, it seems that high pathogen load generates Treg cells both by inducing inflammation as well as directly, thereby suppressing the host effector immune response. Little is known about the frequency dynamics of Treg cells during therapy in human tuberculosis [35]. Our data of therapy induced decline of Treg cells is indicative of their pathogen driven expansion, at least in part among tuberculosis patients. Failure of similar decline among patients with MDR tuberculosis strongly suggests that i) the observed enrichment of Treg cells among patients is driven by the persistent pathogen load, at least partially and declines with successful clearance of bacilli ii) Treg cell monitoring may be an important predictive biomarker for MDR tuberculosis and response to the anti-tuberculosis chemotherapy.

Recent reports clearly indicates Treg cells enrichment during tuberculosis [5], [6], [7], [36]–[39]. However, the role of pathogen driven expansion of Treg cells in human tuberculosis still remains unclear. To address the role of antigen induced specific Treg cells in PTB patients, we directly looked at the frequency of Treg cells following *in vitro* stimulation with *Mtb.* antigen and observed significant antigen driven increase in their frequencies. Moreover, Treg cells were found to be in proliferating cycle both in freshly isolated specimen and following *in vitro* culture. Thus, we conclude that antigen induced Treg cell enrichment occurs among PTB patients and plausibly, heavy pathogen load drives their expansion, thereby, suppressing the host immune response. This seems to be a critical mechanism by which *Mtb.* evades host immunity. This is further supported by recent study of Katara et al on human visceral leishmaniasis [41].

Previously, we demonstrated IL-10 mediated suppression of T cell response among tuberculosis patients [5], Here, we showed PD-1 mediated contact dependent suppression of *Mtb.* specific T cell response. Selective over-expression of PD-1 and PD-1 ligands on Treg cells (and to some extent on effector T cells) encouraged us to investigate its functional impact on the production of IFN-γ against *Mtb.* Blocking PD-1-PD-L1 pathway markedly increased the frequency of antigen specific IFN-γ producing T cells. Moreover, perhaps for the first time we demonstrated that blocking PD-1 expressed on enriched Treg cells restores the production of protective cytokine such as IFN-γ. Interestingly, blocking PD-1 on the Treg cells restored IFN-γ producing NK cells (CD3^−^). Abrogating PD-1 on Treg cells prevented the apoptosis of the antigen specific IFN-γ producing T cells. However, our results do not rule out the suppressive role of PD-1 expressing non-Treg cells (FoxP3-), as Foxp3 staining based sorting of T cells and their use in functional assay is not possible. Therefore, we conclude that PD-1 pathway suppresses the effector T cells involving both Treg and effector T cells. However, significantly higher levels of PD-1 expression on enriched Treg cells strongly suggest the possible role of Treg cells in PD-1 mediated suppression of effector T cell function among PTB patients. Taken together, the present study reaffirms the role of Treg cells and PD-1 molecules preferentially expressed on them in dampening the protective T cell response (IFN-γ biased) and immune pathogenesis of human tuberculosis. PD-1 has been implicated in suppression of immune response in chronic infections such as HIV [15] and HCV [16]–[18]. Javier et al reported restoration of T cell function following PD-1 blockade, suggesting that PD-1 mediated suppression of host immunity plays important role in tuberculosis [19]. Contrastingly, some recent studies of PD-1 knocked out mice demonstrated reduced survival of mice infected with pathologic dose of *Mtb.* [42]–[44]. On the contrary, our results among PTB patients provide strong evidence for the immunosuppressive role of Treg cells and PD-1 expressed on them in the pathogenesis of tuberculosis. Such discordance may be due to aberrantly heightened inflammatory response elicited in PD-1 knocked out animals, which may exert lethal influence on the survival. In fact, those animals showed excessive neutrophillic and necrotic response following infection [42]. In our opinion, these studies do not rule out the role of PD-1 and Treg cells in the suppression of host T cell response during tuberculosis. We propose that basal level of PD-1 expressing Treg cells are required to control the excessive inflammatory response following *Mtb.* infection, as observed among PD-1 deficient mice. On the other hand, over expression of PD-1 on the enriched Treg cells inhibits protective T cell response against *Mtb.* Therefore, inhibiting PD-1 pathway triggered by Treg cells may restore the host immunity and thus help in disease containment.

Recently, it was shown that Treg cells do over express PD-1 and its ligands and their interaction is essential for suppressive activity of Treg cells [23], [24], [45]. Treg cells may suppress the host T cell response by several mechanisms. PD-L1 and PD-L2 has been described to co-stimulate IL-10 production [46]–[47] and expansion of Treg cells [48]. Treg cells also have been reported to inhibit the function of APCs via PD-1 pathway such as down regulation of co-stimulatory molecules CD80 and CD86, leading to inefficient antigen presentation to T cells [45]. Here, we showed that Treg cells can directly inhibit the effector T cells function in PD-1 dependent manner among PTB patients by inducing apoptosis of IFN-γ producing T cells.

In summary, the present study highlights certain crucial aspects of human tuberculosis, which may have potent translational implications. Firstly, Treg cells play important role in suppression of protective T cell response among tuberculosis patients via IL-10 and PD-1 pathway. Secondly, *Mtb.* drives expansion of Treg cells and with their therapeutic clearance Treg cells decline with concomitant rise of antigen specific IFN-γ production. Thirdly, monitoring the frequency of Treg cells may serve as predictive biomarker of MDR tuberculosis. Fourthly, abrogating PD-1 on Treg as well as non Treg cells may restore protective T cell response among tuberculosis patients. This may constitute modalities for adjunct therapeutic and vaccination protocols. Recently, it has been demonstrated that attenuation of Treg cells along with BCG vaccination has a positive impact on the protective immunity against tuberculosis. Therefore, while designing a vaccine for boosting immunity against *Mtb.* repairing of Immune deficit by inhibiting the Treg cells and PD-1 expressed on them may be critical in rescuing protective immunity [49].

## Materials and Methods

### Study cohort

The study was carried out in 21 PTB patients {mean age 29.67±10.65 (range 19–54) years with 15 males and 6 females}. These patients attended the All India Institute of Medical Sciences Hospital, New Delhi, India for diagnosis and treatment. 19 Healthy Controls {HCs, mean age 26.53±6.69 (range 23–47) with 15 males and 4 females} subjects were also included in the study. These HCs are attendants of indoor patients subjects and didn't suffer from any disease and their peripheral blood examination and chest radiograph were normal. All HCs recruited in this study were tuberculin reactive/PPD+. Written informed consent was obtained from all study subjects. The research project was approved by the office of The Ethics Sub-Committee, All India Institute of Medical Sciences, New Delhi, INDIA (**Ref. No.A-60:/25:07:2007**). Diagnosis of tuberculosis was made according to the previously described criteria [5]. Details of diagnostic work-up are summarized in **Table-S1**. All subjects were HIV negative. 8–10 ml of peripheral blood was collected from each PTB patients and HCs. None of the patients was on anti-TB treatment at the time of enrolment.

### Cell isolation and flow cytometry

Cells isolation and flow cytometry performed as previously reported [5]. Freshly isolated PBMCs were surface stained using α-CD4, α-CD25, α-Ki67, α-PD-1, α-PD-L1 and α-PD-L2 antibodies (BD Biosciences and eBiosciences, CA, USA) following staining protocol as per the manufacturer's instructions and were acquired in BD FACS Calibur and analyzed using flowjo software.

### *In vitro* cell culture

To measure *Mtb.* antigen (WCL, 20 µg/ml, JALMA, Taj Ganj, Agra, India) specific intracellular cytokines production and *Mtb.* antigen driven expansion of CD4^+^ Foxp3 Treg cells, freshly isolated PBMCs were cultured (2×10^6^cells/ml) in presence of *Mtb.* antigen for 24 hours {monensin/Golgi transport blocker was added (2 µM, Sigma, USA) during last 6 hours of culture} and 48 hours for *ex vivo* Treg cells expansion. Cultured cells were stained for α-CD4 Cy5PE followed by intracellular detection of α-IL-4 PE, α-IFN-γ FITC and with α-Ki67 PE (BD Biosciences, CA, USA) and α-Foxp3 FITC (eBiosciences, CA, USA). Stained cells were acquired in BD FACS Calibur and analyzed using flowjo software.

### Treg sorting, PD-1-PD-L1 blocking and effector T cell functions assay

Freshly isolated PBMCs were cultured with or without blocking antibodies against PD-1 (5 µg/ml, J116; eBioscience), and/or PD-L1 (5 µg/ml, MIH1; eBioscience) or isotype control, in the presence of *Mtb.* antigen (WCL, 20 µ/ml) with Brefeldin A (10 µg/ml SIGMA, St Louis, Missouri, USA). To demonstrate the role of PD-1:PD-L1 in Treg mediated suppression of Teff cells, CD25^+^ cells were sorted from freshly isolated PBMCs using MACS columns (Miltenyi Biotec, Bergisch Gladbach, Germany). Treg enriched cells were then treated with blocking mAbs to α-PD-1, α-PD-L1 and then mixed at 1∶2 with the enriched T cells and stimulated with *Mtb.* antigen with Brefeldin A (10 µg/ml) for 72 hours and intracellular IFN-γ and IL-2 was measured with flow cytometry. Additionally, *Mtb.* stimulated PBMCs were cultured with or without blocking antibodies against PD-1 and its ligand(s) were stained to determined apoptosis of *Mtb.* antigen reactive (IFN-γ producing) T cells by annexinV and caspase-3 staining. Stained cells were acquired in BD FACS Calibur and analyzed using flowjo software.

#### ELISA for IL-10

Sandwich ELISA for soluble IL-10 (Cat. No. 555157 BD Pharmingen, San Diego, CA, USA) were performed as per manufacturer instructions in the culture supernatants obtained from different time points of PBMCs culture under *Mtb.* antigen stimulation.

### Statistical analysis

All statistical analyses were performed with both SPSS v 11.5 (SPSS Inc .Chicago, IL) and Prism software (GraphPad version 5; La Jolla, CA) using Pearson's correlation test, Wilcoxon rank sum test and Mann-Whitney U test. Differences were considered significant at P<0.05.

## Supporting Information

Figure S1 **Analysis of** ***Mtb.*** **antigen (WCL) specific cytokine producing CD4 T cells by flow cytometry.** Freshly isolated PBMCs of PTB patients prior to anti-tubercular treatment were cultured for 24 hours with *Mtb.* antigen (WCL) and with PMA (5 ng/ml sigma, USA), Ionomycin (2 mM, Sigma, USA) used as positive control against *Mtb.* antigen in presence of golgi transport inhibitor Brefeldin A. Cultured cells were washed and surface stained with anti-CD4 followed by IFN-γ and IL-4 intracellular staining. Percentage of IFN-γ and IL-4 producing cells were analyzed on gated CD4^+^ T cells. FACS plots shows 3 representative cases, each from PTB patients (A, upper panel) and Healthy Controls (B, lower panel) along with positive controls (PMA and Ionomycin).
(DOC)

Figure S2 **Longitudinal analysis of frequencies of Foxp3^+^ Treg cells gated on CD4^+^CD25^+^ cells during anti-tubercular treatment and post therapy among PTB patients.** The percentages of CD4^+^CD25^+^Foxp3^+^ Treg cells was determine in 16 PTB patients 4 MDR TB patients at various time point (Day0-before commencement of therapy and at 3 month, 6 month and 12 month). (**A**) Representative histogram plot showing gradual decline in CD4^+^CD25^+^Foxp3^+^ Treg cells in the peripheral blood of 3 PTB patients during and after post therapy. (**B**) Horizontal line graph showing CD4^+^CD25^+^Foxp3^+^ Treg cells among 16 PTB patients at various time points. Statistical analysis of values was performed with the parametric paired t-test, two-tailed. Each line represents a single individual.
(DOC)

Figure S3 **Measurement of soluble interleukine-10 (IL-10) levels in** ***Mtb.*** **antigen specific (WCL)** ***c*** **ulture supernatants.** Scatter plot showing measurement of soluble IL-10 by ELISA in *Mtb.* antigen specific (WCL) culture supernatants obtained from PBMCs of PTB patients at various time points (Day0, 1month, 3month, 6month and 12month). At each time point freshly isolated PBMCs derived from PTB patients (n = 21) cultured with *Mtb.* antigen (WCL) for 24 hours and culture supernatants were utilized for IL-10 ELISA. Soluble IL-10 levels significantly decline with time point. Statistical analysis of values was performed with the parametric paired t-test, two-tailed. Each symbol represents a single individual.
(DOC)

Table S1 **Demographic and clinical details of the Pulmonary Tuberculosis Patients.**
(DOC)
