# Supplementary material for: Foxp3+ Regulatory T Cells among Tuberculosis Patients: Impact on Prognosis and Restoration of Antigen Specific IFN-γ Producing T Cells
Source: PLoS One. 2012 Sep 19;7(9):e44728. doi: 10.1371/journal.pone.0044728 (PMC3446959; doi:10.1371/journal.pone.0044728)
Supplement: Figure S1 — Analysis of Mtb. antigen (WCL) specific cytokine producing CD4 T cells by flow cytometry. Freshly isolated PBMCs of PTB patients prior to anti-tubercular treatment were cultured for 24 hours with Mtb. antigen (WCL) and with PMA (5 ng/ml sigma, USA), Ionomycin (2 mM, Sigma, USA) used as positive control against Mtb. antigen in presence of golgi transport inhibitor Brefeldin A. Cultured cells were washed and surface stained with anti-CD4 followed by IFN-γ and IL-4 intracellular staining. Percentage of IFN-γ and IL-4 producing cells were analyzed on gated CD4+ T cells. FACS plots shows 3 representative cases, each from PTB patients (A, upper panel) and Healthy Controls (B, lower panel) along with positive controls (PMA and Ionomycin). (DOC) [file pone.0044728.s001.doc]

**Figure S1: Representative FACS plots showing *Mtb.* antigens (WCL) specific cytokines (IFN-γ and IL-4) among PTB patients and Healthy Controls**

**A**

**B**

**Figure S1**: **Analysis of *Mtb.* antigen (WCL) specific cytokine producing CD4 T cells by flow cytometry.** Freshly isolated PBMCs of PTB patients prior to anti-tubercular treatment were cultured for 24 hours with *Mtb.* antigens (WCL) and with PMA (5ng/ml sigma, USA), Ionomycin (2mM, Sigma, USA) used as positive control against *Mtb.* antigen in presence of golgi transport inhibitor Brefeldin A. Cultured cells were washed and surface stained with anti-CD4 followed by IFN-γ and IL-4 intracellular staining. Percentage of IFN-γ and IL-4 producing cells were analyzed on gated CD4^+^ T cells. FACS plots shows 3 representative cases, each from PTB patients (A, upper panel) and Healthy Controls (B, lower panel) along with positive controls (PMA and Ionomycin).
