# Supplementary material for: Foxp3+ Regulatory T Cells among Tuberculosis Patients: Impact on Prognosis and Restoration of Antigen Specific IFN-γ Producing T Cells
Source: PLoS One. 2012 Sep 19;7(9):e44728. doi: 10.1371/journal.pone.0044728 (PMC3446959; doi:10.1371/journal.pone.0044728)
Supplement: Figure S2 — Longitudinal analysis of frequencies of Foxp3+ Treg cells gated on CD4+CD25+ cells during anti-tubercular treatment and post therapy among PTB patients. The percentages of CD4+CD25+Foxp3+ Treg cells was determine in 16 PTB patients 4 MDR TB patients at various time point (Day0-before commencement of therapy and at 3 month, 6 month and 12 month). (A) Representative histogram plot showing gradual decline in CD4+CD25+Foxp3+ Treg cells in the peripheral blood of 3 PTB patients during and after post therapy. (B) Horizontal line graph showing CD4+CD25+Foxp3+ Treg cells among 16 PTB patients at various time points. Statistical analysis of values was performed with the parametric paired t-test, two-tailed. Each line represents a single individual. (DOC) [file pone.0044728.s002.doc]

**Figure S2: Longitudinal analysis of Foxp3^+^Treg cells gated on CD4^+^CD25^+^ cells during anti-tubercular treatment and post therapy among PTB patients**

**A**

**B**

**Figure S2**: **Longitudinal analysis of frequencies of Foxp3^+^Treg cells gated on CD4^+^CD25^+^ cells during anti-tubercular treatment and post therapy among PTB patients.** The percentages of CD4^+^CD25^+^Foxp3^+^ Treg cells was determine in 16 PTB patients 4 MDR TB patients at various time point (Day0-before commencement of therapy and at 3 month, 6 month and 12 month). **(A**) Representative histogram plot showing gradual decline in CD4^+^CD25^+^Foxp3^+^ Treg cells in the peripheral blood of 3 PTB patients during and after post therapy**. (B)** Horizontal line graph showing CD4^+^CD25^+^Foxp3^+^ Treg cells among 16 PTB at various time points. Statistical analysis of values was performed with the parametric paired t-test, two-tailed. Each line represents a single individual.
