# Supplementary material for: Foxp3+ Regulatory T Cells among Tuberculosis Patients: Impact on Prognosis and Restoration of Antigen Specific IFN-γ Producing T Cells
Source: PLoS One. 2012 Sep 19;7(9):e44728. doi: 10.1371/journal.pone.0044728 (PMC3446959; doi:10.1371/journal.pone.0044728)
Supplement: Figure S3 — Measurement of soluble interleukine-10 (IL-10) levels in Mtb. antigen specific (WCL) c ulture supernatants. Scatter plot showing measurement of soluble IL-10 by ELISA in Mtb. antigen specific (WCL) culture supernatants obtained from PBMCs of PTB patients at various time points (Day0, 1month, 3month, 6month and 12month). At each time point freshly isolated PBMCs derived from PTB patients (n = 21) cultured with Mtb. antigen (WCL) for 24 hours and culture supernatants were utilized for IL-10 ELISA. Soluble IL-10 levels significantly decline with time point. Statistical analysis of values was performed with the parametric paired t-test, two-tailed. Each symbol represents a single individual. (DOC) [file pone.0044728.s003.doc]

**Figure S3: Measurement of soluble interleukine-10 (IL-10) levels in *Mtb.* antigens specific (WCL) *c*ulture supernatants obtained from PBMCs of PTB patients during anti-tubercular treatment and post therapy**

**Figure S3**: **Measurement of soluble interleukine-10 (IL-10) levels in *Mtb.* antigens specific (WCL) *c*ulture supernatants**. Scatter plot showing measurement of soluble IL-10 by ELISA in *Mtb.* antigens specific (WCL) culture supernatants obtained from PBMCs of PTB patients at various time points (Day0, 1month, 3month, 6month and 12month). At each time point freshly isolated PBMCs derived from PTB patients (n=21) cultured with *Mtb.* antigen (WCL) for 24 hours and culture supernatants were utilized for IL-10 ELISA. Soluble IL-10 levels significantly decline with time point. Statistical analysis of values was performed with the parametric paired t-test, two-tailed. Each symbol represents a single individual.
