# Supplementary material for: Foxp3+ Regulatory T Cells among Tuberculosis Patients: Impact on Prognosis and Restoration of Antigen Specific IFN-γ Producing T Cells
Source: PLoS One. 2012 Sep 19;7(9):e44728. doi: 10.1371/journal.pone.0044728 (PMC3446959; doi:10.1371/journal.pone.0044728)
Supplement: Table S1 — Demographic and clinical details of the Pulmonary Tuberculosis Patients. (DOC) [file pone.0044728.s004.doc]

**Table-S1: Demographic and clinical details of the Pulmonary Tuberculosis (PTB) Patients**

| **No. of PTB patients** | 21 |
| --- | --- |
| **Demographic characteristics** | |
| Age (Mean ± SD, range) | 29.67± 10.65 (range 19-54) years |
| Sex (M/F) | 15/6 |
| BMI (Kg/m^2^, mean± SD) | 17.5± 2.80 |
| Ethnicity | India |
| **Clinical details** | |
| **Radiological classification**  **Extent of lesion-Chest Radiograph** | **No. of cases** |
| Minimal lesions | 2 |
| Moderately advanced lesions | 13 |
| Far advanced lesions | 6 |
| Unilateral | 8 |
| Bilateral | 13 |
| Cavitary (Yes/No) | 19/2 |
| Number |  |
| Three | 2 |
| Two | 6 |
| One | 11 |
| **Bacillary load (sputum positivity)** | **No. of cases** |
| 3+ | 8 |
| 2+ | 6 |
| 1+ | 7 |
| Mantoux test (positive/negative) | 17/4 |
| **Diagnostic characteristics** | |
| **Smear for Mycobacterium tuberculosis** | **No. of cases** |
| Sputum | All |
| **Culture for Mycobacterium tuberculosis**  {L-J Medium or BACTEC (460)} | **No. of cases** |
| (positive/negative) | 12/9 |

Abbreviations: J medium= Lowenstein-Jensen medium, TB=tuberculosis
